# Effect of open versus closed endotracheal suctioning on intracranial pressure in severe brain‐injured children: Study protocol for a randomized controlled trial

**DOI:** 10.1002/nop2.656

**Published:** 2021-05-26

**Authors:** Yan Li, Xiaoyan Li, Zunjia Wen, Xin Zhang, Yingfei Liu, Li Wei

**Affiliations:** ^1^ SICU Children’s Hospital of Nanjing Medical University Nanjing City China

**Keywords:** brain injury, children, intracranial pressure, mechanical ventilation; airway, suction

## Abstract

**Aim:**

To compare the effects and safety of open and closed endotracheal suction in children with severe brain injury.

**Design:**

A single‐blinding, single‐centre randomized controlled trial (RCT).

**Methods:**

The children with severe brain injury admitted to the intensive care unit (ICU) from 1 September 2020–31 August 2022 will be included. And a total of 172 children with severe brain injury are expected to be included. The intracranial pressure, SpO_2_ and heart rate before suctioning, at the end of suction, and at 5 and 10 min after suction, the estimated sputum volume for each suction, the incidence of ventilator‐associated pneumonia, the duration of mechanical ventilation and the length of ICU stay will be analysed.

**Results:**

This present RCT has been prospectively registered in China Clinical Trial Registry (http://www.chictr.org.cn, ChiCTR2000030963). This present study is expected to provide reliable evidence to the airway management in children with severe brain injury.

**Abbreviations**
ICP: intracranial pressure
ICU: intensive care unit
RCT: randomized controlled trial
*SD*
: standard deviation
SICU: surgical intensive care unit
VAP: ventilator‐associated pneumonia

## 1 BACKGROUND

Brain injury is a common serious problem with extremely high mortality and disability rates in clinical settings, which is very common in car or fall accidents (Capizzi et al., 2020). It has been reported that the incidence of traumatic brain injury ranks the second among the trauma of various body organs, but the mortality of traumatic brain injury ranks the first (Dewan et al., 2016). Previous studies (Cheng et al., 2017; Taylor et al., 2017) have reported that the mortality of patients with moderate‐to‐severe brain injury in the paediatric intensive care unit (ICU) can be up to 41.9%. The brain injury not only poses a serious threat to the life and health of patients, but also places a heavy economic and spiritual burden on their families and society (Steinbuchel et al., 2020; Uski et al., 2018). Children with brain injury are often accompanied by fluctuations in intracranial pressure (ICP) (Bennett et al., 2017). Those patients with severe brain injuries are prone to complications such as cerebral hernia, epilepsy and even death with regard to the disturbed intracranial pressure (Delaplain et al., 2020; Svedung Wettervik et al., 2020). Therefore, for children with severe brain injury, maintaining the stability of ICP is of great importance for the prognosis of patients.

Mechanical ventilation is an important way of life support for children with severe brain injury, and the airway management during mechanical ventilation plays a key role. Intratracheal suction is an important nursing operation for the management of mechanical ventilation in children (American Association for Respiratory Care, 2010). Currently, there are two kinds of methods for sputum suctions in patients with mechanical ventilation, namely open endotracheal suction and closed endotracheal suction (Afshari et al., 2014). The open sputum suction is a traditional method used in clinical practice. It has the advantages of relatively simple operation, less time‐consuming and effective, but it also has the disadvantages of aerosol exposure and interruption of mechanical ventilation (Faradita Aryani & Tanner, 2018). Different from the open endotracheal suction, the closed endotracheal suction is a kind of method developed in the last decades. It can perform sputum suction operation while ensuring the operation of mechanical ventilation, avoiding the detachment of the breathing circuit (Kuriyama et al., 2015; Letchford & Bench, 2018). To some extent, it ensures the tightness of the airway tubing, which is beneficial to maintain the oxygenation of patients, and it can reduce the risk of nursing staff being exposed to the aerosol during the suction process (Hamishekar et al., 2014). Many scholars (Copnell et al., 2007; Faraji et al., 2015; Mohammadpour et al., 2015) have evaluated the effects and safety of open suction and closed suction, but their results are inconsistent.

It is worth noting that the current research on the role of open and closed sputum suction and ICP is mostly limited to the population of adults or newborns (age < 28 days), but very few in the population of children with age < 18 years. How does sputum suction affect ICP in children with severe brain injury and mechanical ventilation? Is there any difference in the effect and safety of open and closed endotracheal suction in children with severe brain injury? These issues need to be further evaluated. Therefore, it is necessary to compare the effects and safety of open and closed endotracheal suction in children with severe brain injury, thereby providing evidence to support the airway management of children with mechanical ventilation.

## 2 METHODS

### 2.1 Study design

**Figure 1:**
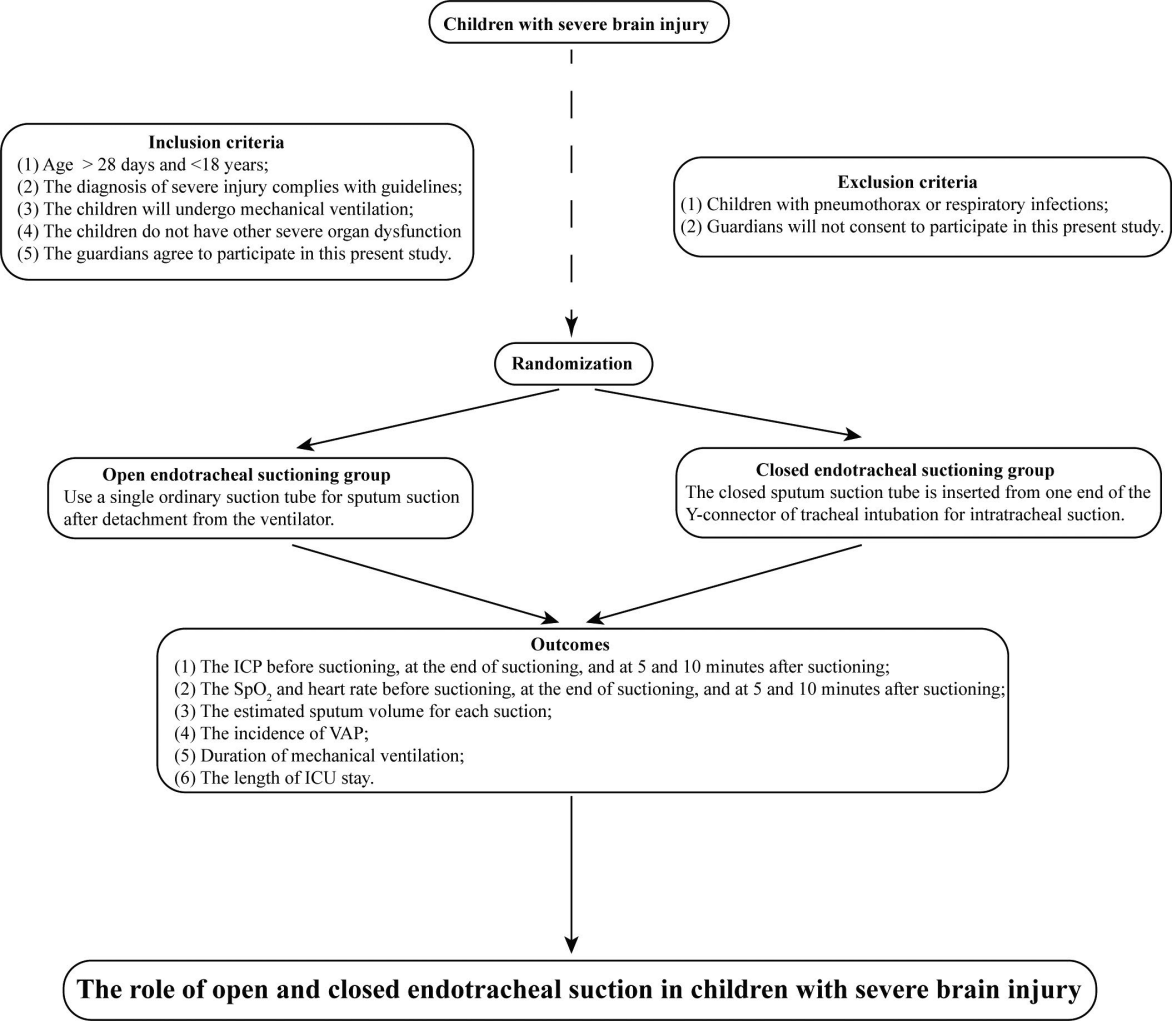
The flow chart of study design

This study is designed as a single‐blinding, single‐centre randomized controlled trial (RCT), and the flow chart of study design is presented in Figure 1. We will perform blinding to the included patients, but we do not apply the blinding to the interventionists and the outcome observers.

### 2.2 Ethical considerations

This present study has been approved by the ethics committee of our present hospital (202001008–1). Any other significant potential risks and advantages will be informed to the parents and healthcare providers. And written informed consents will be obtained from all the included children or their relatives.

### 2.3 Trial status

This present RCT has been registered in China Clinical Trial Registry (http://www.chictr.org.cn, ChiCTR2000030963). We have registered it on 20 March 2020. We are intended to recruit the participants on 1 September 2020, and recruitment will be completed approximately on 31 August 2022.

### 2.4 Participants

The children with severe brain injury admitted to the surgical ICU (SICU) in our hospital from 1 September 2020 to 31 August 2022 will be considered for inclusion.

The inclusion criteria are as follows: (a) the age of children should be >28 days and <18 years; (b) the diagnosis of severe brain injury should comply with the relevant guidelines (Carney et al., 2017; Gao, 2017); (c) the children will undergo mechanical ventilation, and all the participants will be under the 24‐hr cerebral protection; (d) the children do not have other severe organ dysfunction, such as congenital heart disease; and (e) the guardian or relatives will be informed and agree to participate in this present study.

The exclusion criteria are as follows: (a) children with pneumothorax, atelectasis or other serious congenital diseases or respiratory infections; and (b) children or the guardians will not consent to participate in this present study and sign the informed consent.

### 2.5 Sample size calculation

We calculate the sample size with the following formula for the rate comparison of two groups (Sakpal, 2010):

$$n=\frac{\lambda }{2{\left(\arcsin\sqrt{P_{\max}}‐\arcsin\sqrt{P_{\min}}\right)}^{2}}$$

We assume α = 0.05, β = 0.2 and ν = 3–1 = 2, then λ = 8.84, and we set *P*_max_ and *P*_min_ as the maximum and minimum fluctuation rates of during sputum suction in our pre‐trials (54.6% and 20.9%, respectively), then we come to the result of *n* ≈ 78. Furthermore, considering that the attrition rate of about 10% of the study participants, the number of patients included in each group should be at least 86. Therefore, a total of 172 patients are intended to be included.

### 2.6 Randomization and allocations

With reference to related reports, we are intended to use the method of random number table (Brocklehurst & Hoare, 2017) to randomly divide the included candidates into open endotracheal suctioning group and closed endotracheal suctioning group. We have aimed to blinding staff and patient intervention selection at the point of randomization, but once the intervention occurs then nurses can no longer be blinded. And the nurse will conduct the open and closed endotracheal suction accordingly.

### 2.7 Blindness

We are going to only set blindness on patients with similar suction equipment, limited by the number of nursing staff, and we will not set blindness on the nurses during intervention and outcome assessments.

### 2.8 The suction intervention

In this study, the open and closed endotracheal suction will be performed accordingly in two groups. Before suctioning, both groups of children will undergo the same dose of pure oxygen. And chest percussions will be performed before suction. All the patients will be put in supine position during suction since the change of position can affect the ICP, and the stableness of ICP is vital to the management of TBI patients. The open suctioning group will use a single ordinary suction tube (Pacifi, China) for sputum suction after detachment from the ventilator, while for the closed endotracheal suction group, a specific tube (Geri, China) for sputum suction will be used. That is, the closed sputum suction tube was inserted from one end of the Y‐connector of tracheal intubation for intratracheal suction. Suction will be performed whenever patient needs, and the suction pressure used will be controlled under 80–100 mmHg. The duration of every suction is intended to be less than 15 s, and the suction can be proceeded until obtaining the sputum within 15 s.

### 2.9 Outcome assessment

The outcomes collected and analysed in this present study are as follows: (a) the ICP (mmHg) level before suctioning, at the end of suctioning, and at 5 and 10 min after suctioning; (b) the pulse oxygen saturation (SpO_2_,%) and heart rate (times/min) before suctioning, at the end of suctioning, and at 5 and 10 min after suctioning; (c) the estimated sputum volume (ml) for each suction; (d) the incidence of ventilator‐associated pneumonia(VAP): the diagnosis and treatment of VAP will be in comply with related guidelines (Ardehali et al., 2020; Liapikou et al., 2019; Shi et al., 2019); (e) the duration of mechanical ventilation (days); and (6) the length of ICU stay (days).

### 2.10 Statistical analysis

SPSS 23.0 software will be used for data analysis in the present study. The enumeration data will be expressed with mean ± standard deviation (*SD*), and the categorical variable data will be expressed with percentages (%). Test for normality will determine whether to use parametric test (independent *t* test/paired *t* test) or (Mann–Whitney test/ Wilcoxon's test). In this present study, *p* < .05 will be considered statistically significant.

## 3 RESULTS

Our study is planned to commence on 1 September 2020 and is expected to run until 31 August 2022, with a total duration of 24 months. We will use the intention‐to‐treat analysis to retain the randomized information as much as possible, and we will compare and adjust the characteristics to minimize the homogeneity of participants. We have conducted a pilot study with four patients to check the feasibility of the study, and it is feasible in our department. And based on the results of pilot study, we expected that the closed suctions may have more advantages in stabilizing the ICP level, and it may have the equal effects in the suctioned sputum volume and preventing the VAP. The results are expected to be shared in August 2022.

## 4 DISCUSSION

Mechanical ventilation plays a key role in life support for children with severe head injury, and the airway management during mechanical ventilation is essential to the prognosis of patients (Fraga Gomes Martins et al., 2019). Intratracheal sputum suction is an important part in the management of children with mechanical ventilation, which acts to keep the children's airway open by removing the sputum or secretions in the airway (Coppadoro et al., 2019). If the suction cannot remove the sputum of patients in a timely and effective manner, it will not only affect the oxygenation function of children, but also colonize a large number of sputum and bacteria in the lungs and increase the risk of VAP (Dexter & Scott, 2019; Rouze et al., 2018). However, it has been reported (Fraga Gomes Martins et al., 2019) that the suction during the mechanical ventilation may also lead to secondary brain injury. Therefore, how to ensure the effectiveness and safety of sputum suction is on the top research agenda of respiratory managements in children with mechanical ventilation.

Open suction and closed suction are the two of most commonly seen approaches of suction clinically. Hao et al. (Hao & Wang, 2018) have conducted a meta‐analysis on the effectiveness and safety of closed suction and open suction in adults, and a total of 9 RCTs have been included, and the results have shown that compared with open endotracheal suction, the closed endotracheal suction can prevent the occurrence of VAP and reduce the duration of mechanical ventilation, but it cannot reduce the mortality. It is worth noting that the quality of RCTs included in this study is limited, thereby the results obtained have limited insights into clinical setting. Meanwhile, the previous study (Lu et al., 2018) has shown that the frequency of changing the closed suction device has a significant effect on the colonization of bacteria at the tip of the suction tube. At present, there are still controversies (Shamali et al., 2019; Williams et al., 2018) about the advantages and disadvantages of closed and open endotracheal suction in the incidence of VAP, mortality and length of hospital stay. Therefore, there are still many controversies over the role of open and closed endotracheal suction in clinical practice, and further researches are needed.

ICP monitoring plays an important role in the treatment and prognosis of patients with brain injury. Several previous studies (Argent, 2019; Chen et al., 2019; Miles et al., 2020) have shown that there is a significant correlation between the changes in ICP and cerebral blood flow perfusion with various treatment and nursing procedures. The nursing operation of suction can lead to transient increase in ICP, but its long‐term effects remain unclear. Giancarlo et al (Galbiati & Paola, 2015) have systematically analysed 14 related studies and have pointed out that although the risk of ICP increasing above 20mm Hg during open suction is higher, it is still unclear which suction approach is more conducive to the maintenance of ICP and brain blood perfusion balance. Therefore, further research is needed to determine the best suction technique for nursing practice.

Several limitations must be considered in this present study. Firstly, even though we have organized a research team to collect the data, we cannot ensure all the normal nursing staff will follow the procedures as we designated, we cannot ensure data are collected on every suction episode, especially for those patient requires emergency suctioning, and the data may not be collected timely. We will conduct several trainings on the details of our procedure to ensure the accuracy of collected data. Secondly, sample size is not large in this present study, and there may be some differences in the suction pressure among different populations, and future studies with larger sample size and multi‐centre are needed.

To the best of our knowledge, there are very few studies focused on the role of open and closed endotracheal suctions and potential effects on the ICP in the children population. In this present study, we have aimed to assess the potential effects of open and closed endotracheal suction on ICP in the population of children, to provide credible evidence to the nursing care of airway. The results of this study are expected to provide basis for the suction practice of airway management in children.

## 5 ETHICS APPROVAL AND CONSENT TO PARTICIPATE

This present study has been approved by the Ethics Committee of Children's Hospital of Nanjing Medical University (202001008–1). And written informed consents will be obtained from all the included children or relatives.

## CONFLICT OF INTEREST

No conflict of interest has been declared by the authors.

## AUTHOR CONTRIBUTIONS

Y L and L W designed research; Y L, X L, Z W and X Z conducted research; Z W and Y L analysed data; Y L wrote the first draft of the manuscript; and Z W and L W had primary responsibility for final content. All authors read and approved the final manuscript.

## Data Availability

All the data will be available and reported in our future manuscript with regard to this study protocol, and we are willing to share the data to scholars who are interested in this issue whenever necessary.
